# The efficacy and safety of half-dose glucocorticoids combined with rituximab versus high-dose glucocorticoids for initial treatment of minimal change disease: a single-center experience

**DOI:** 10.3389/fphar.2024.1403562

**Published:** 2025-01-06

**Authors:** Xueting Li, Peng Yan, Lu Zhang, Wei Qiao, Zhengbiao Xue, Xiangdong Fang, Ben Ke, Shuying Zhu

**Affiliations:** ^1^ Department of Nephrology, The Second Affiliated Hospital, Jiangxi Medical College, Nanchang University, Nanchang, Jiangxi, China; ^2^ Department of Healthy Center, Nanchang Normal University, Nanchang, Jiangxi, China; ^3^ Department of Intensive Care Unit, The First Affiliated Hospital of Gannan University, Ganzhou, Jiangxi, China

**Keywords:** minimal change disease, glucocorticoids, rituximab, adverse reaction, nephrotic syndrome

## Abstract

**Background:**

Minimal change disease (MCD) is a podocytopathy more commonly seen in children, but it also accounts for 10%–25% of adult nephrotic syndrome. High-dose oral glucocorticoids were recommended for initial treatment of MCD. However, long-term use of systemic corticosteroids is associated with significant adverse events, such as steroid-induced diabetes and infections. The aim of this study was to investigate the clinical efficacy and safety of half-dose glucocorticoids combined with rituximab (RTX) for the initial treatment of MCD.

**Methods:**

We recruited 74 patients with MCD confirmed by renal biopsy. Twenty patients were treated with RTX alone with 1000 mg at d1 and d15, 28 patients received half-dose prednisolone (0.5 mg/kg) per day combined with RTX with 1000 mg at d1, and 26 patients received high-dose prednisolone (1 mg/kg) per day. Treatment responses, including complete remission (CR) and partial remission (PR), and outcome adverse events such as steroid-induced diabetes and infections were compared among the three groups after 12 months of follow-up.

**Results:**

At the 12-month follow-up, the CR rates were 50%, 96.4%, and 96.2% for the RTX group, half-dose prednisolone combined with RTX group, and high-dose prednisolone group, respectively. There was no statistical difference between the half-dose prednisolone combined with RTX group and high-dose prednisolone group on CR and PR and kidney function (*P* > 0.05). Compared with the high-dose prednisolone group, the half-dose prednisolone combined with RTX group had a reduced incidence of adverse events of steroid diabetes (*P* = 0.041), especially in patients older than 55 years of age.

**Conclusion:**

The efficiency of half-dose prednisolone combined with RTX is not inferior to the recommended treatment regimen, and this regimen can effectively reduce the incidence of steroid-induced diabetes in patients with MCD. Moreover, we recommend a half-dose prednisolone combined with RTX treatment for elderly patients with MCD.

## Introduction

Minimal change disease (MCD) is an important cause of nephrotic syndrome in adults, accounting for 10%–15% of cases (Kidney Disease: Improving Global Outcomes Glomerular Diseases Work Group, 2021). High-dose oral glucocorticoids were recommended for initial treatment of MCD (Kidney Disease: Improving Global Outcomes Glomerular Diseases Work Group, 2021). However, long-term use of systemic corticosteroids is associated with significant adverse events, such as steroid-induced diabetes and infections, which can be fatal, especially in the elderly. Rituximab (RTX) is a chimeric IgG1 monoclonal antibody targeting CD20, capable of depleting CD20 pre-B cells and mature B cells for at least 6–12 months. Observational studies have shown that RTX is effective in treating frequently relapsing or steroid-dependent MCD in patients needing glucocorticoids, with or without other maintenance immunosuppressive therapies (Ruggenenti et al., 2014; Guitard et al., 2014; Iwabuchi et al., 2018; Munyentwali et al., 2013). Overall, the efficacy of RTX in inducing remission is between 65% and 100%, and notably, it is associated with a reduction in the number of relapses immunosuppressive medications (Kidney Disease: Improving Global Outcomes Glomerular Diseases Work Group, 2021). However, experience with RTX is limited, and the long-term efficacy and risks in the initial treatment of MCD are unknown. The aim of this study is to investigate the clinical efficacy and safety of half-dose glucocorticoids combined with RTX for initial treatment of MCD.

## Methods

This study followed the guidelines of the Declaration of Helsinki and was approved by the Ethics Committee of the Second Affiliated Hospital of Nanchang University, in addition to informed consent from each patient.

### Study design

**FIGURE 1:**
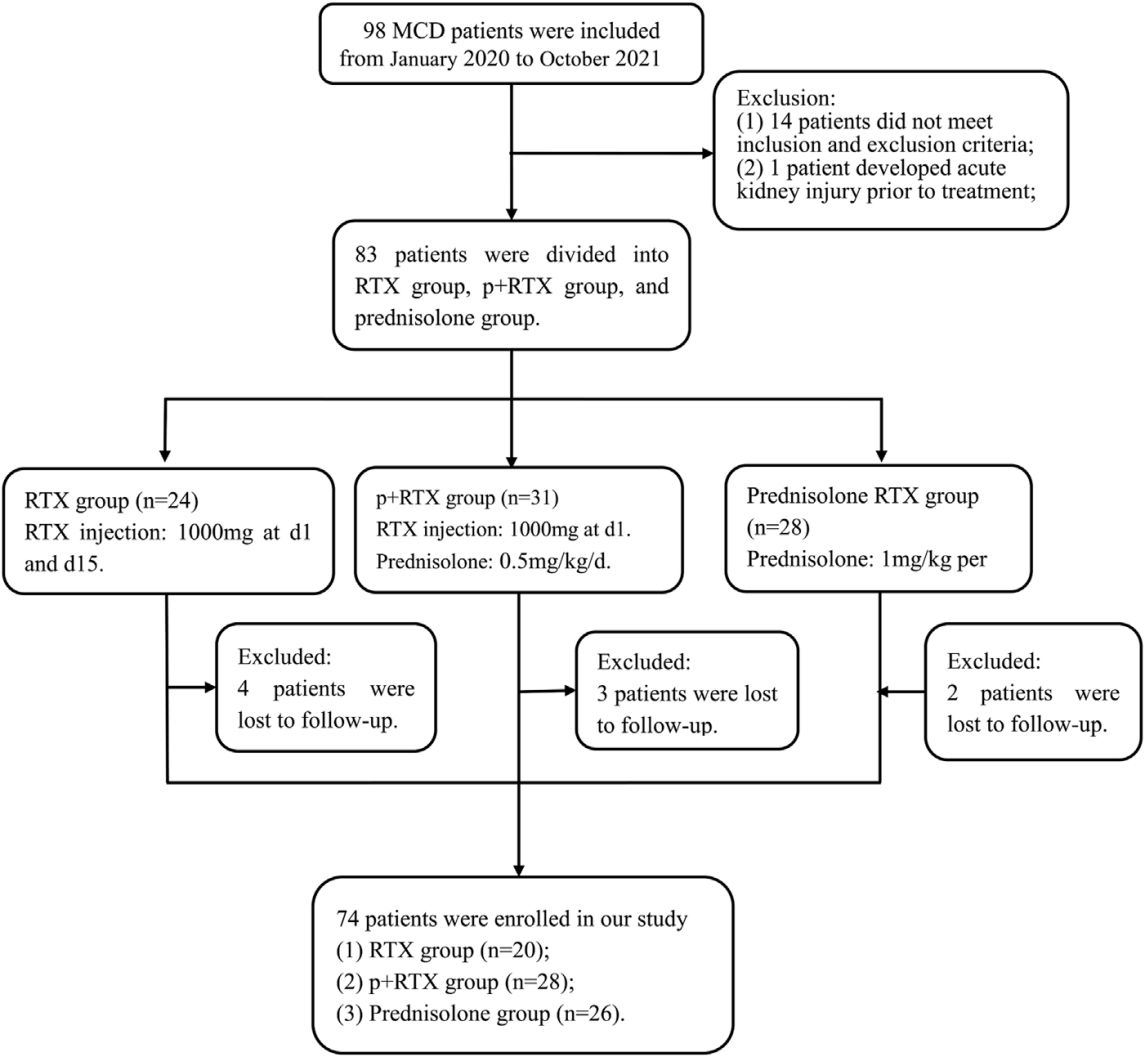
Flowchart of the study.

In this single-center retrospective study, 98 patients with biopsy-proven MCD diagnosed in the Department of Nephrology of the Second Affiliated Hospital of Nanchang University were recruited from January 2020 to October 2022 (Figure 1). The inclusion criteria were as follows: (1) age >18 years old; (2) MCD diagnosed by renal biopsy within 24 months of the first immunotherapy; (3) urinary protein >3.5 g/d; (4) estimated glomerular filtration rate (eGFR) > 45 mL/min/1.73 m2 (CKD-EPI equation).

The exclusion criteria were as follows: (1) presence of active infection; (2) combined with type 1 or 2 diabetes mellitus; (3) poorly controlled hypertension (>140/90 mmHg); (4) being pregnant or breastfeeding; (5) severe hepatic dysfunction or cardiovascular disease; (6) having received steroid or immunosuppressive therapy within the last 3 months; (7) history of tumor.

### Intervention

The half-dose prednisolone combined with RTX regimen (p+RTX) consists of daily oral prednisolone (0.5 mg/kg) combined with RTX with 1000 mg at d1. The RTX regimen (RTX) consists of 1,000 mg of RTX on d1 and d15. The high-dose prednisolone regimen (prednisolone) consists of daily oral prednisolone (1 mg/kg). The maximum dose of hormones we use is 60 mg. The glucocorticoid dose was tapered 2 weeks after complete remission according to the latest 2021 KDIGO guidelines (Kidney Disease: Improving Global Outcomes Glomerular Diseases Work Group, 2021).

### General clinical parameters

Clinical data were collected from medical records at the time of patient diagnosis and every 1–3 months of follow-up. Baseline data were obtained prior to immunotherapy and included demographic and histological characteristics of all patients and laboratory indices, including age, sex, blood pressure, proteinuria, serum albumin, creatinine, and eGFR (calculated by the CKD-EPI formula (Levey et al., 2009)).

All patients were followed-up for at least 12 months. Follow-up ended at the last visit or when the patient reached end-stage renal disease or died.

### Outcome indicators

Primary outcomes included complete remission (CR) or partial remission (PR) within 12 months. CR: urine protein ≤0.3 g/24 h; ALB ≥35 g/L; normal renal function. PR: urine protein of 0.3–3.5 g/24 h or >50% reduction from baseline; ALB ≥35 g/L; stable renal function. Total remission (TR) was defined as either CR or PR. The composite remission encompassed CR or PR. Time to remission was defined as the time from study entry to remission and was measured primarily at the second week and months 3, 6, 9, and 12.

We regarded adverse events, such as infections, infusion reactions, leukopenia, and steroid-induced diabetes, as secondary clinical outcomes.

Relapse was defined as the reappearance of proteinuria exceeding 3.5 g/24 h following the patient’s attainment of either CR or PR.

In assessing renal function, the presence of renal dysfunction was defined as a reduction in eGFR exceeding 50% from the baseline.

### Statistical analysis

Continuous variables are expressed as mean ± SD or median (Q25; Q75), depending on the distribution of the variable. Categorical variables were described using counts and percentages. Student’s t-test or ANOVA was employed for continuous variables, while the χ2 test or Fisher exact test was utilized for categorical variables. Cumulative complete remission rates and composite remission rates among the three groups were estimated using the Kaplan–Meier method and assessed using the log-rank test. Logistic regression was used to explore predictors of treatment-acquired infectious events. *P* values <0.05 were considered statistically significant, and all tests performed were two-tailed unless otherwise stated. All statistical analyses for the study were performed using SPSS version 22.0 (SPSS Inc., Chicago, Illinois, United States).

## Results

### Baseline characteristics

Seventy-four patients with MCD were recruited in this study, which included 20 patients who were treated with RTX alone with 1000 mg at d1 and d15, 28 patients who received half-dose prednisolone (0.5 mg/kg) per day combined with RTX with 1000 mg at d1, and 26 patients who received high-dose prednisolone (1 mg/kg) per day.

Baseline demographic characteristics and renal function indices were similar in each group. Specifically, in the initial phase, no significant differences were detected in age, gender, proteinuria, albumin, Scr, blood pressure, and eGFR levels among the included patients (Table 1).

**TABLE 1:** Baseline characteristics of MCD patients in three groups.

Characteristics	RTX	Half-dose prednisolone combined with RTX group	High-dose prednisolone group	*P*
Number of patients	20	28	26	
Age (years)	45 (28, 69)	42.25 (22, 66)	30.875 (19, 72)	0.746
Gender, n (%)				0.871
Males	10 (50%)	11 (39%)	13 (50%)	
Females	10 (50%)	17 (61%)	13 (50%)	
BP (mmHg)				
SBP	119.7 ± 10.0	122.2 ± 11.3	127.3 ± 11.6	0.127
DBP	80.4 ± 12.2	82.0 ± 11.6	82.6 ± 12.7	0.866
Proteinuria (g/24 h)	8.67 (3.91, 18.62)	10.75 (7.33, 16.7)	8.78 (4.13, 17.4)	0.531
ALB (g/L)	23.77 (16.6, 28.6)	20.63 (16.57, 23.7)	20.88 (11.49, 28.32)	0.16
Scr (μmol/L)	81.24 ± 25.19	78.95 ± 38.93	81.23 ± 27.95	0.983
eGFR (ml/min/1.73 m^2^)	90.5 ± 17.7	94.9 ± 23.2	92.7 ± 19.2	0.783

ALB, albumin; BP, blood pressure; SBP, systolic blood pressure; DBP, diastolic blood pressure; scr, serum creatinine; eGFR, estimated glomerular filtration rate; *P* < 0.05 was statistical difference.

### Primary outcomes

#### The CR and PR

**FIGURE 2:**
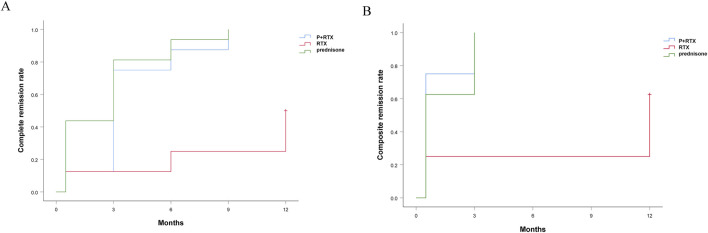
Cumulative complete remission rate and cumulative composite remission rate in the three groups during follow-up. Red line: Rituximab; Blue line: Hormone + rituximab; Green line: Hormones alone.

There were no significant differences between p+RTX and high-dose prednisolone in the proportion of patients in CR and PR. The treatment efficacy was evaluated on the basis of the outcome of patients who completed 9 months of therapy. After 3 months of initial therapy, 4 (20%), 20 (71%), and 17 (65%) cases achieved CR in RTX, p+RTX, and high-dose prednisolone groups, respectively (*P* = 0.06) (Figure 2A). At 9 months, CR was experienced by 5 of 20 patients (25%) in the RTX group, 27 of 28 patients (96.4%) in the p+RTX group, and 25 of 26 patients (96.2%) in the high-dose prednisolone group (Figure 2B). However, at 12 months, the number of patients who experienced CR in the RTX group was increased to 10 of 20 patients (50%), and CR in the other two groups was similar with 12 months (Figure 2B). Except the RTX alone group, median times to CR or TR were similar at 3 months in the p+RTX and high-dose prednisolone groups, respectively. No recurrence was reported in the p+RTX and high-dose prednisolone groups.

### Second outcomes

#### Urinary protein level

**FIGURE 3:**
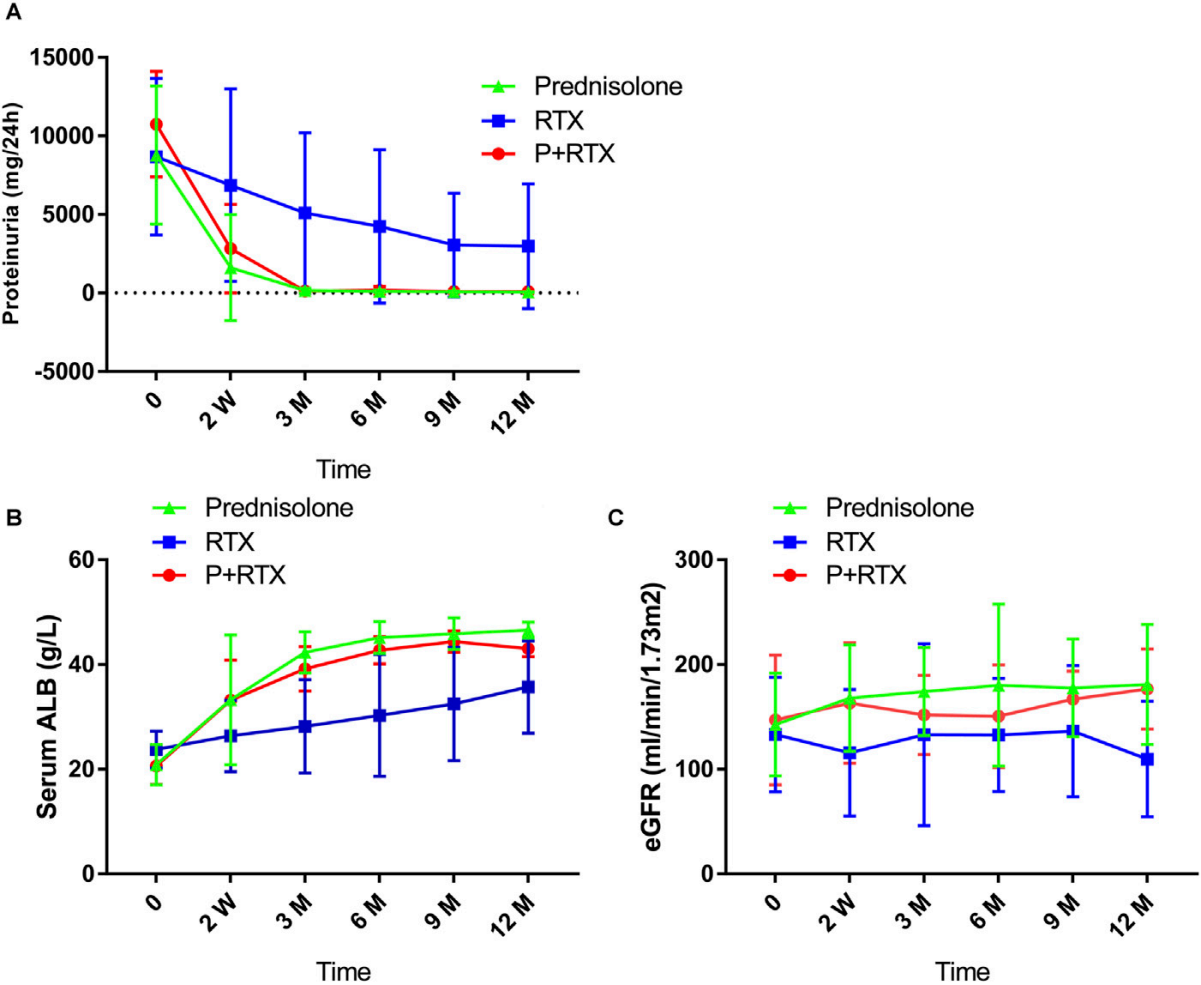
Changes in clinical indicators during follow-up in the three groups. **(A)** 24-h urinary protein; **(B)** serum albuminuria (ALB); and **(C)** estimated glomerular filtration rate (eGFR).

At 12 months after initial therapy, the 24-h urinary protein decreased from 8.67 ± 4.98 g to 2.974 ± 3.958 g in the RTX group (*P* = 0.0239) and from 10.75 ± 3.36 g to 0.09 ± 0.03 g in the p+RTX group (*P* < 0.001), as well as from 8.78 ± 4.39 g to 0.05 ± 0.03 g in the high-dose prednisolone group (*P* < 0.001) (Figure 3A). The reduction in proteinuria was similar between the p+RTX and high-dose prednisolone groups (66.4% ± 9.86% in the p+RTX group and 75.4% ± 6.73% in the high-dose prednisolone group, *P* = 0.351). There was no statistically significant difference between these two groups in the level of urinary protein excreted through all observation times (*P* > 0.05). In the p+RTX and high-dose prednisolone groups, a significant decline in proteinuria levels was observed in the patients, starting from the first 2 weeks.

#### Serum albumin level

The level of serum albumin increased from 20.88 ± 3.84 g/L to 46.58 ± 1.49 g/L in the high-dose prednisolone group (*P* < 0.001) and from 20.63 ± 3.57 g/L to 43.01 ± 1.51 g/L in the p+RTX group (*P* < 0.001) at the end of 12 months. Meanwhile, the RTX monotherapy only increased albumin levels from 23.77 ± 3.53 g/L to 35.72 ± 8.83 g/L (*P* < 0.001) (Figure 3B). The serum albumin level in the p+RTX and high-dose prednisolone group was significantly increased compared with that of the RTX alone group at 3–12 months (*P* = 0.018 at 3 months, *P* = 0.018 at 6 months, *P* = 0.016 at 9 months, *p* = 0.037 at 12 months), whereas there was no significant difference between the p+RTX and high-dose prednisolone groups during all the observations (*P* > 0.05).

#### Kidney function

The initial serum creatinine level was 81.24 ± 25.19 umol/L, 78.96 ± 38.93, and 81.23 ± 27.95 umol/L, and final level was 103.54 ± 42.84 mmol/L, 59.01 ± 8.90, and 62.9 ± 12.48 umol/L, respectively, in patients who were treated with RTX, p+RTX, and high-dose prednisone (Figure 3C). Improvement in the plasma creatinine level was observed in the patients after 2 weeks of treatment in the p+RTX and high-dose prednisone groups. At the end of the study, plasma creatinine decreased to normal level in all patients (100%) from the p+RTX and high-dose prednisone groups and 15 patients (75%) from the RTX group. However, there were no significant differences in the serum creatinine level between the p+RTX group and the prednisone group.

### Adverse events

Adverse events were recorded from the initial therapy. No patient experienced new onset of serious adverse events in the RTX alone group. Therefore, we primarily listed the occurrence of adverse events between the other two groups. The difference in the incidence of infusion reactions and infections between the two groups was not statistically significant (*P* = 0.086 and *P* = 0.375, respectively) (Table 2). Furthermore, there was no significant difference in the incidence of steroid-induced diabetes between the two groups (*P* = 0.095). Interestingly, an age subgroup analysis was conducted, revealing that patients aged 55 years or older had significantly higher rates of steroid-induced diabetes in the prednisone group compared to the p+RTX group (*P* = 0.041).

**TABLE 2:** Comparison of the incidence of adverse events (AEs) between the two groups.

Event	p+RTX (N = 28)	Prednisone (N = 26)	*P*
Reaction/drug intolerance	4 (14.3)	0 (0)	0.086
Infections	3 (10.7)	5 (19.2)	0.379
Steroid diabetes n (%)	1 (3.5)	5 (19.2)	0.095
Age >55, N	12	8	
Age >55 steroid diabetes	0 (0)	3 (37.5)	**0.041**
Death	0 (0)	0 (0)	

*P*-value of Fisher’s exact test.

Bold value represents *p* value less than 0.05.

## Discussion

In this study, we examined the effects of half-dose prednisolone in combination with RTX in patients with MCD covering all age groups and compared the effect to those patients receiving high-dose prednisolone as well as those receiving RTX alone. The results showed that the addition of half-dose prednisolone to RTX was consistent with the remission rate and the level of urinary protein decline in the traditional high-dose prednisolone regimen and resulted in fewer serious adverse events requiring clinical intervention during the 12-month follow-up period. Certainly, this regimen is significantly more effective than RTX alone, causing a reduced risk of serious side effects. Thus, our data suggest that half-dose prednisolone therapy with additional RTX combination has an antiproteinuric and side-effect-reducing effect in patients with MCD.

MCD is the most common primary nephrotic syndrome in children but also accounts for 15% of adult nephrotic syndrome (Waldman et al., 2007). Glucocorticoids are recommended as the first-line therapy for MCD by the Kidney Disease: Improving Global Outcomes (KDIGO) (Kidney Disease: Improving Global Outcomes Glomerular Diseases Work Group, 2021). Long-term steroid therapy comes with adverse clinical effects such as steroid-induced hyperglycemia, increased salt and water retention leading to hypertension and increased cardiovascular events, dyslipidemia, decreased bone mineralization, and psychiatric disease (Liu et al., 2013). These side effects limit its clinical therapeutic limitations, and there is an urgent need for alternatives that can achieve both the remission rates associated with adequate amounts of hormones and the reduction of steroid-associated side effects.

RTX is currently the first-line treatment option for moderate- and high-risk primary membranous nephropathy (Fervenza et al., 2019). In addition, RTX is an option for treating patients with frequently relapsing or steroid-dependent MCD and focal segmental glomerulosclerosis (FSGS), lupus nephritis, ANCA-associated small-vessel vasculitis renal damage, and membranoproliferative glomerulonephritis (Chan et al., 2020; Ramos-Casals et al., 2011). However, for renal diseases such as MCD and FSGS, RTX is generally not recommended as an initial treatment regimen. Rather, RTX can be considered for patients with high relapse rate, and since traditional immunosuppressive regimens require a relatively long duration of use and their continued use is not recommended given the cumulative toxic side effects, RTX may be considered (Hogan and Radhakrishnan, 2013). Several studies have focused on the efficacy of RTX in patients with frequently recurring MCD (Hansrivijit et al., 2020), but the efficacy of first-time use of half-dose steroid combined with RTX in primary MCD has not previously been demonstrated.

The kidney-protective effects of RTX in MCD treatment have been well established (Webendorfer et al., 2020; Długosz et al., 2022; Kannan, 2022). Recently, a random-controlled trial also reported an improvement in remission rates, duration of remission, in children with steroid-dependent nephrotic syndrome nephropathy with RTX (Ravani et al., 2013). Thus, we hypothesized that the improvement effects of RTX might be a potential add-on therapy for adult patients with MCD. We administered RTX to patients with primary MCD but still had a lower relapse rate than traditional corticosteroid therapy. We found that the CR at 3rd month and PR at 6th month cal remission rate both reached the same plateau in the p+RTX group and in the prednisone group. More importantly, the rate of steroid-induced hyperglycemia was significantly lower in the p+RTX group for patients older than 55 years of age. During the 12-month follow-up, the clinical remission rate was consistent at all times in the p+RTX group and in the prednisone group. All of these findings provide strong evidence of the efficacy and safety of optimized rituximab regimens.

Additionally, proteinuria was significantly reduced and serum albumin gradually increased in the p+RTX group and the high-dose prednisone group after immunosuppressive therapy. Of note, after 2 weeks of treatment from the initial period, there was no significant difference observed in these two groups, suggesting that p+RTX might be not inferior to classical prednisone throughout the whole duration of the treatment. This finding may be related to the role for B cells in addition to T cells in the pathogenesis of MCD (Sinha and Bagga, 2013).

Our study showed that RTX alone had a 50% complete response rate for MCD, which was similar to previous studies. Nan Guan et al. reported the efficacy of RTX in MCD initiation therapy. They found that RTX alone had a complete response rate of only 55.6% for MCD (Guan et al., 2023). It is well known that RTX works mainly by targeting the CD20 antigen expressed by B cells and reducing the associated immune response such as antibodies produced by B cells, while MCD has long been considered a T-cell-mediated disease. Frequent relapses or corticosteroid-dependent patients treated with RTX all relapse after recovery from RTX-induced peripheral B-cell depletion (Iijima et al., 2022), but in some patients, CD19^+^ B cells are not detected, but MCD relapses, and patients continue to use RTX and go into remission again (Guan et al., 2023), so how RTX plays a role in MCD is still not fully known. This may interfere with both autoreactive T cells and B cells to induce MCD remission, but there may be individualized factors in the patient. Impaired regulatory T cell (Treg) function in patients with MCD has been reported, and Treg cells have been found to induce remission of nephrotic syndrome (Araya et al., 2009; Le Berre et al., 2009). Meanwhile, previous studies have shown that RTX maintains remission in patients with nephrotic syndrome due to the restoration of Treg cell function, and they found that RTX may enhance the number and function of Treg cells (Stasi et al., 2008). In patients with MCD, the number of Treg and the levels of Treg-related cytokines (TGF-β1 and IL-10), and transcription factor (Foxp3) significantly increased after corticosteroid treatment and closely correlated with disease activity (Liu et al., 2011). These studies suggest that both circulating B cells and T cells are involved in the development of MCD, but there can still be individualized factors in the treatment process. The complete remission rate of low-dose hormone combined with RTX in MCD can reach 100% (Sun et al., 2024). Therefore, we recommend half-dose hormone combined with RTX to treat MCD, which not only has a high complete remission rate but also has fewer side effects.

P+RTX treatment was safe and well tolerated. Side effects occurred in one of new-onset glucocorticoid-induced diabetes mellitus (3.5%) and three of infection (10.7%) patients, which were not severe and mostly resolved by effective therapy. Kidney function was stable during the entire follow-up period in both P+RTX and prednisone treatment groups.

The main limitations of this study are the relatively small single-center sample and the lack of randomization. The follow-up time in our trial was relatively short, and lack of window of observation for statistical recurrence rates was observed.

## Conclusion

This study is the first to report that RTX alone is not effective in the initial treatment of MCD. Our data are also the first to support that the efficacy of half-dose prednisolone in combination with RTX therapy is not inferior to that of the sufficient prednisolone treatment. Meanwhile, this treatment is more satisfactory in terms of safety in reducing side effects for elderly patients with MCD. The long-term efficacy and safety of p+RTX need future randomized controlled trials.

## Data Availability

The original contributions presented in the study are included in the article/Supplementary Material; further inquiries can be directed to the corresponding authors.
